# Spin Interactions in Supramolecular Assemblies of Porphyrin Oligomer Radical Anions

**DOI:** 10.1002/anie.202518425

**Published:** 2025-10-07

**Authors:** Janko Hergenhahn, Sebastian M. Kopp, Henrik Gotfredsen, Kan Tang, Stephen Barlow, Seth R. Marder, Christiane R. Timmel, Harry L. Anderson

**Affiliations:** ^1^ Chemistry Research Laboratory Department of Chemistry University of Oxford 12 Mansfield Road Oxford OX1 3TA U.K; ^2^ Centre for Advanced Electron Spin Resonance Department of Chemistry University of Oxford Oxford OX1 3QR U.K; ^3^ Renewable and Sustainable Energy Institute University of Colorado Boulder Boulder CO 80309 USA; ^4^ Departments of Chemical and Biological Engineering and of Chemistry University of Colorado Boulder Boulder CO 80309 USA

**Keywords:** Diradical, Dipolar coupling, EPR spectroscopy, Porphyrin, Supramolecular chemistry

## Abstract

Diradicals have potential applications in spintronics, molecular electronics, and dynamic nuclear polarization due to their unique electronic structure. Supramolecular diradicals are particularly attractive, as changes in conformation can be used to switch the spin state. In this work, we explore a class of supramolecular diradicals formed by the self‐assembly of charged zinc porphyrin oligomers with bidentate ligands such as 1,4‐diazabicyclo[2.2.2]octane (DABCO) and 4,4'‐bipyridyl. Field‐dependent nutation experiments on the anions of porphyrin ladder complexes show nutation frequencies corresponding to doublet and triplet species, which can both be attributed to the same spin system using simulations based on the time evolution of the density matrix. Density functional theory was used to calculate dipolar coupling tensors and provided insights into the electronic structure of these double‐stranded assemblies.

## Graphical abstract

Double‐strand porphyrin ladder complexes offer an excellent way to hold two spin centers in a well‐defined structure. Nutation experiments on the dianion of such complexes confirm the presence of doublet and triplet species. Spin dynamics simulations show that the emergence of these higher multiplicities can be fully accounted for by considering the dipolar interaction between electron spins.

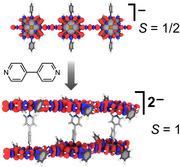

The two unpaired electrons in a diradical may interact intramolecularly via exchange coupling *J* and/or dipolar coupling *D*.^[^ ^1^, ^2^ ^]^ When the unpaired electrons are sufficiently far apart, the interactions are small compared to the (difference in) Zeeman interactions and only result in small perturbations of the energy levels of the uncoupled spin wavefunctions. However, if the interactions are large, they result in a mixing of the spin levels and formation of singlet and triplet states, the relative energies of which depend on the sign and magnitude of *J* and *D*. Diradicals have potential applications in organic field‐effect transistors and near‐infrared (NIR) organic photodetectors due to their small effective bandgap resulting from the small energy difference between singlet and triplet states.^[^ ^3^ ^]^ The possibility of switching between different spin states also makes them attractive for memory devices, spintronics, and logic gates for quantum computing^[^ ^4^ ^]^ and supramolecular chemistry can provide an additional handle for controlling the spin states of diradical species.^[^ ^5^, ^6^, ^7^, ^8^, ^9^ ^]^ In addition, diradicals have also found applications in dynamic nuclear polarization (DNP) to enhance the sensitivity of NMR measurements.^[^ ^1^, ^10^, ^11^ ^]^

Here, the aggregation behavior of the radical anions of a butadiyne‐linked porphyrin trimer **P3** will be explored. Butadiyne‐linked porphyrin oligomers with 3,5‐bis‐(octyl‐oxy)phenyl groups have previously been found to form discrete π‐stacked aggregates in their neutral states.^[^ ^12^, ^13^ ^]^ Reduction of **P3** to its radical anion might be expected to result in stronger aggregation, as found with many extended π‐systems such as tetracyanoquinodimethane (TCNQ).^[^ ^14^, ^15^ ^]^ Such aggregates have only small exchange coupling interactions between the unpaired electrons due to the lack of covalent connection^[^ ^16^ ^]^ but they can give rise to significant dipolar couplings due to the proximity of the π‐interfaces. The distance between π‐systems and the resulting dipolar coupling can be controlled by formation of well‐defined porphyrin ladder complexes (as shown in Figure 1). Using field‐dependent nutation experiments and numerical simulations, we show that due to the small magnitude and orientation dependence of the dipolar coupling, these systems can give rise to both doublet and triplet spin multiplicities.

**Figure 1:**
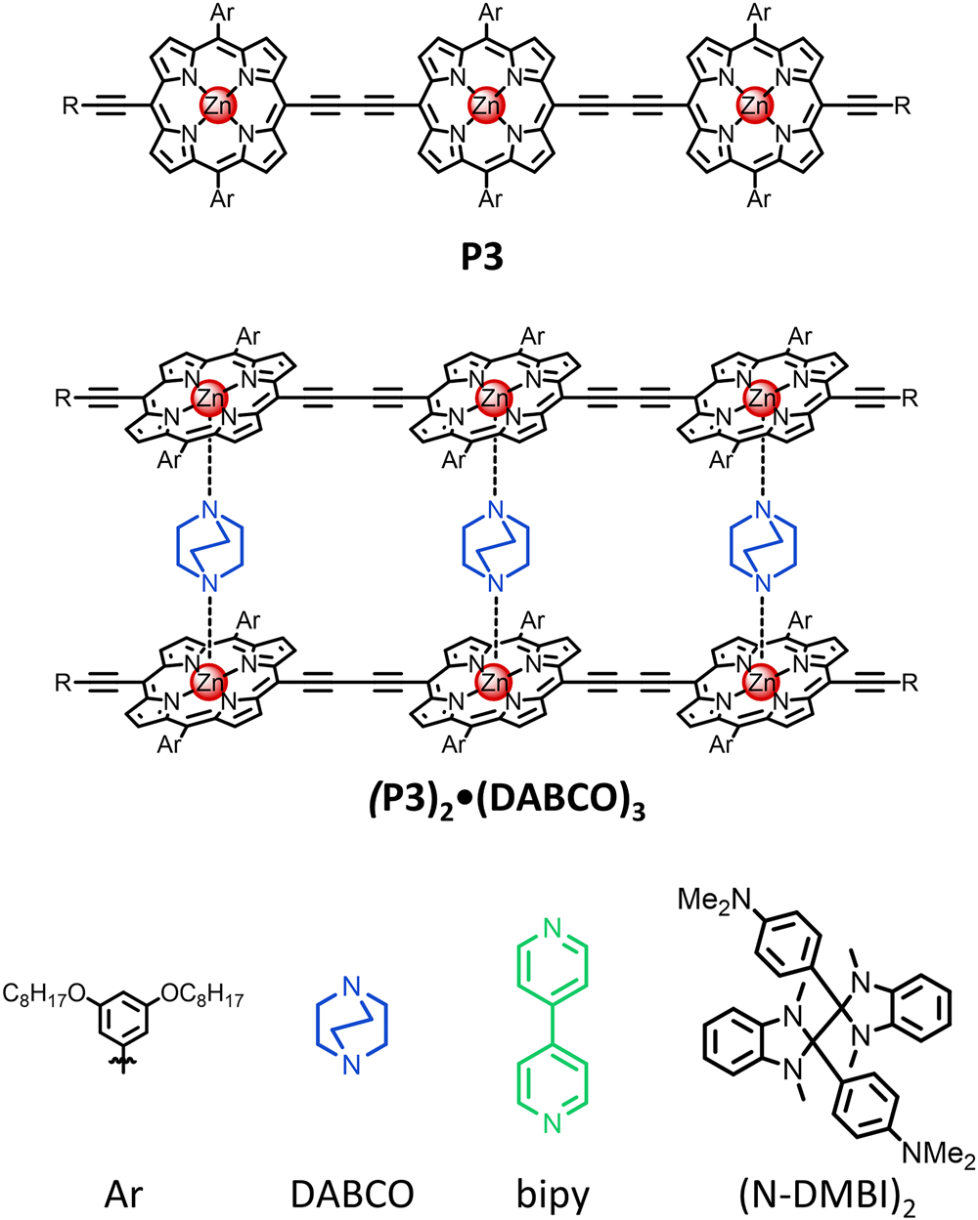
Chemical structures of butadiyne‐linked porphyrin trimer **P3** with 3,5‐bis(octyloxy)phenyl solubilizing groups and DABCO ladder complexes **(P3)_2_•(DABCO)_3_**. R = Si(i‐Pr)_2_(CH_2_)_3_CN.

The radical anion of the trimer **P3** was produced by reduction with the two‐electron reducing agent (N‐DMBI)_2_ in toluene, which has a suitable effective reduction potential (*E* ≈ −2 V vs. ferrocene Fc/Fc^+^)^[^ ^17^, ^18^ ^]^ and was found to only result in the singly reduced **P3^•−^**, without over‐reduction to the dianions or trianions (see Figure S1). In addition, the resulting counter ion N‐DMBI^+^ does not form tight ion‐pairs with the reduced porphyrins, unlike other reducing agents that were tested (see Figure S2). The echo‐detected field‐sweep spectra of solutions of **P3^•−^** in toluene with different concentrations are shown in Figure 2a. A stoichiometric amount of reducing agent was used in each case. The spectrum recorded at 50 µM is narrow and resembles that of the previously studied radical anion of the porphyrin trimer with bulky 3,5‐bis(trihexylsilyl)phenyl groups that are known to prevent aggregation.^[^ ^19^ ^]^ At higher concentrations, the signal becomes significantly broadened out. Particularly at 800 µM, an additional shoulder appears at higher field values.

**Figure 2:**
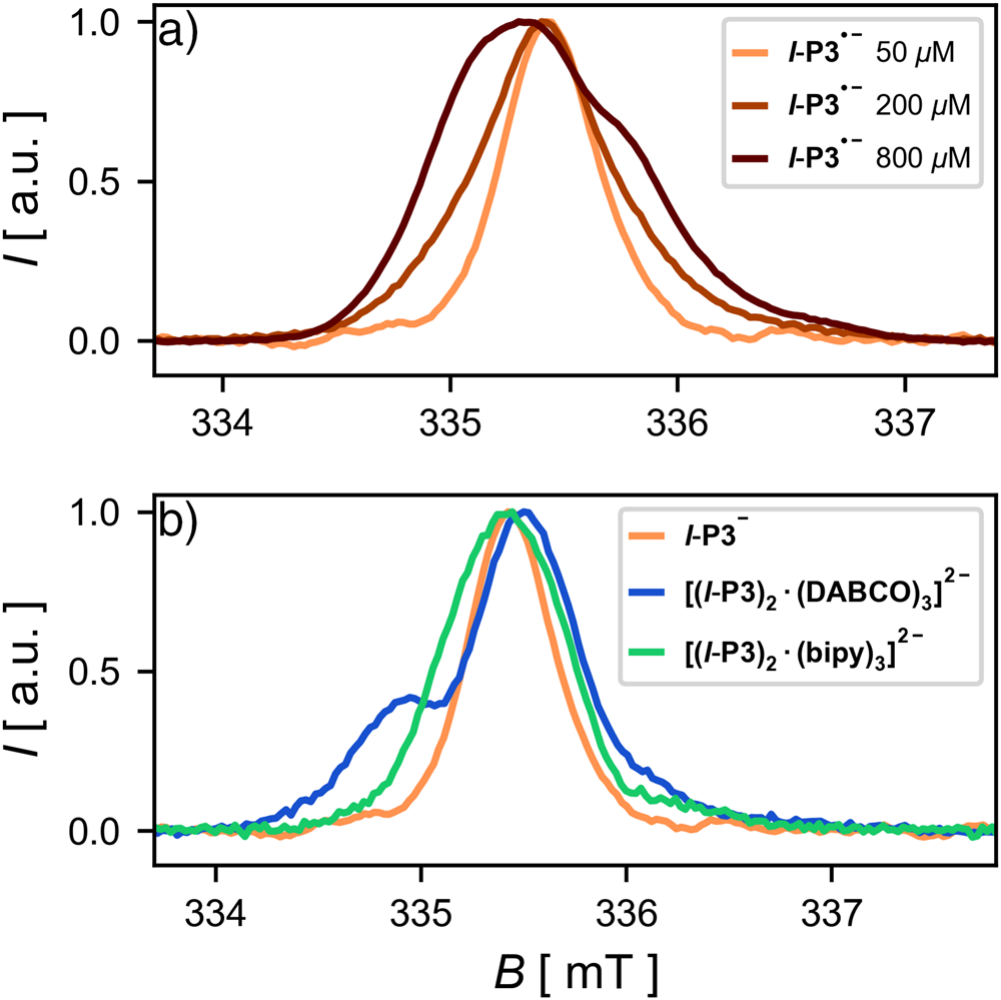
Echo detected field sweep spectra of a) **P3^•−^** solutions with different concentrations and b) **P3^•−^** ladder complexes with a **P3** concentration of 50 µM recorded at 80 K at X‐band frequencies in toluene, generated using (N‐DMBI)_2_. The spectra were aligned to the same excitation frequency (9.40 GHz) and scaled to the same intensity to facilitate comparison of their spectral shapes.

Echo‐detected transient nutations recorded at the shoulders of the spectra at *B* = 334.8 mT (for 200 and 800 µM) showed more than one nutation frequency, which indicates the presence of more than one spin multiplicity.^[^ ^20^ ^]^ To improve on the relatively poor resolution that was obtained from the echo‐detected transient nutation, the more advanced phase‐inverted echo‐amplitude detected nutation (PEANUT) experiment was used.^[^ ^21^ ^]^ Figure 3 shows the Fourier transform of the field‐dependent PEANUT spectra recorded for solutions of **P3^•−^** at different concentrations. The PEANUT field sweep recorded at 50 µM only shows a single nutation frequency, which corresponds to the monoanion **P3^•−^** doublet. A slice along the field axis taken through the maximum of the signal results in a field sweep spectrum that is identical in shape to the previously recorded echo‐detected field sweep (see Figure S3). The PEANUT field sweep spectra recorded at 200 and 800 µM both show an additional signal at a higher frequency. The smaller intensity of this signal for lower concentrations indicates that the additional signal is a result of aggregation.

**Figure 3:**
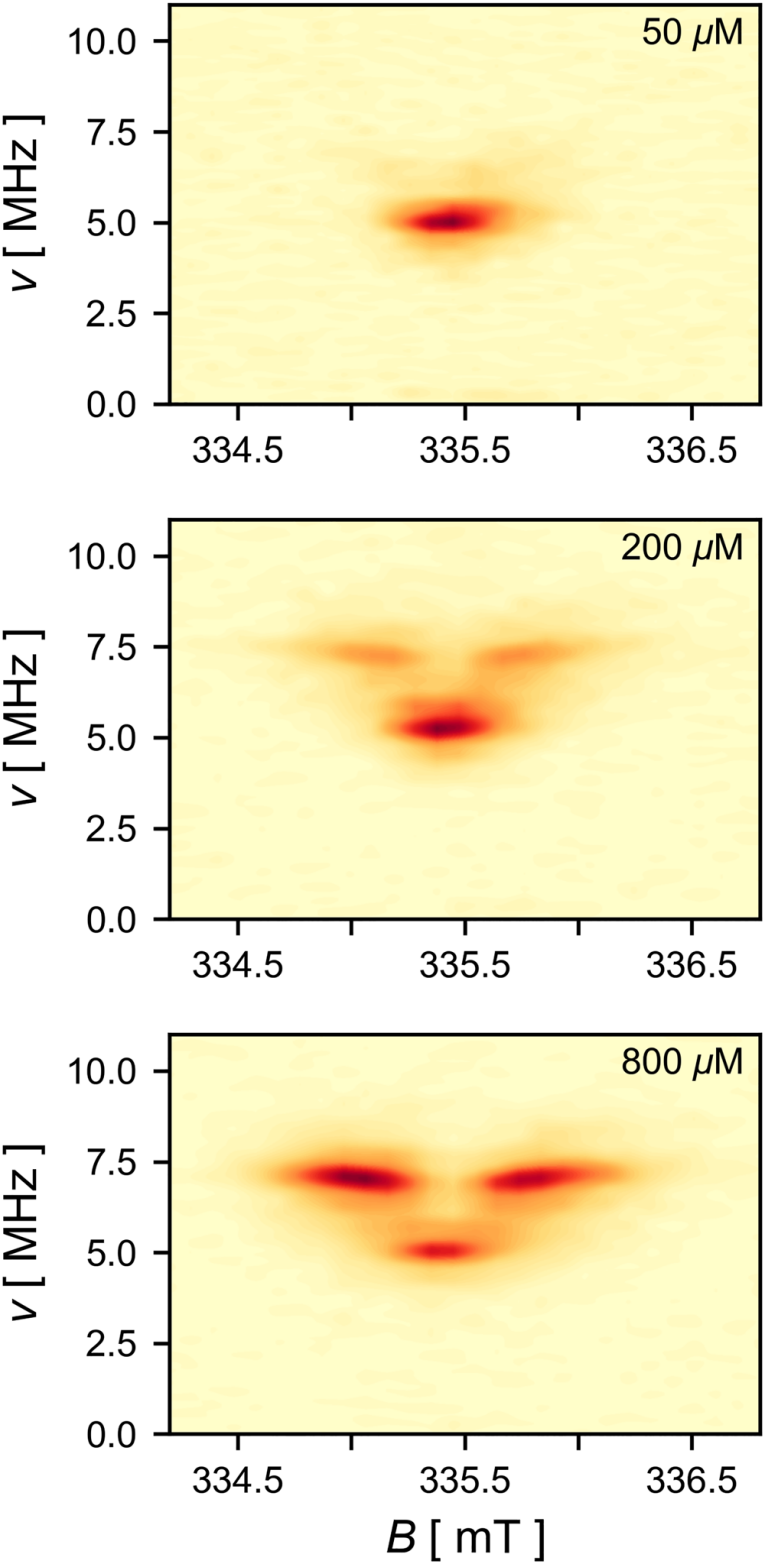
Field‐dependent PEANUT spectra of **P3^•−^** with different concentrations recorded at 80 K at X‐band frequencies in toluene.

The ratio of the frequencies at which the signals appear can provide insights into the spin states that are involved. For the PEANUT field sweep spectrum recorded at a concentration of 200 µM, the two frequencies are 5.28 and 7.29 MHz, corresponding to a ratio of 1.38. For a selective excitation pulse, the nutation frequency *ω*_nut_ of a given transition is:^[^ ^20^ ^]^

(1) $$\omega_{\mathrm{nut}}=\omega_{1}\sqrt{S\left(S+1\right)-m_{S}\left(m_{S}\pm 1\right)}$$

where *ω*_1_ is the microwave field strength in angular frequency units, *S* is the effective spin quantum number, and *m*_*S*_ is the magnetic quantum number of the spin level from which excitation occurs. Therefore, an excitation of a triplet transition leads to a nutation frequency $\sqrt{2}\;\approx \;$1.41 times that of a doublet. This is close to the ratio observed experimentally in the **P3^•−^** aggregate, confirming the presence of a triplet state due to aggregation.

The presence of both doublet and triplet species is likely to arise from an equilibrium between the non‐aggregated and aggregated forms, which is shifted toward the side of the latter as the concentration is increased. A detailed interpretation of the triplet spectrum is difficult, as the molecular structure of the aggregate is not known. Although it is probably similar in form to previously observed π‐stacked interdigitated structures,^[^ ^12^, ^13^ ^]^ calculation of magnetic parameters from EPR seems futile. Instead, the focus was shifted away from the aggregates of free **P3^•−^** and toward well‐defined porphyrin ladder complexes.

Addition of bidentate ligands such as DABCO (1,4‐diaza‐bicyclo[2.2.2]octane) or bipy (4,4'‐bipyridine) to linear butadiyne‐linked porphyrin oligomers is known to give rise to self‐assembly of well‐defined ladder aggregates, in which the porphyrin chains adopt a co‐planar conformation (see Figure 1).^[^ ^22^, ^23^ ^]^ This process can be followed by UV–VIS–NIR spectroscopy, as the co‐planar conformation increases the conjugation between porphyrin units and leads to a red‐shift of the porphyrin Q‐band absorption (see Figures S5 and S6).^[^ ^22^, ^23^ ^]^ This change in the absorption spectrum reaches an end point once 1.5 equivalents of DABCO are added, which confirms the 2:3 stoichiometry of the complex **(P3)_2_(DABCO)_3_**. The isosbestic point indicates that only two species are involved in the equilibrium, the free trimer and the ladder complex; a large excess of ligand is necessary to break up the ladder complex to form the 1:3 complexes, in which only one of the nitrogens of the DABCO ligands is coordinated to zinc.^[^ ^23^ ^]^

The formation of the ladder complexes with both DABCO and bipy was confirmed by UV–VIS–NIR spectroscopy prior to EPR measurements. The ladder aggregates were then reduced by addition of 0.55 equivalents of reducing agent, (N‐DMBI)_2_, in terms of the concentration of **P3**, i.e., 1.1 equivalents of reducing agent in terms of the complex in order to form **[(P3)_2_•(bipy)_3_]^2−^** and **[(P3)_2_•(DABCO)_3_]^2−^**. These solutions were prepared at a concentration of **P3** of 50 µM, which in the case of the free oligomer **P3^•−^** did not result in aggregation or formation of a triplet state. Figure 2b shows the echo detected field sweep spectra of the two ladder complexes and the free trimer as a comparison. Formation of the complex causes similar broadening effects seen before in the more concentrated samples of free **P3^•−^**.

Nutation experiments were again used to probe the multiplicities of the species present in the samples. Both complexes give rise to distinct doublet and triplet signals, which were separated using field‐dependent PEANUT experiments (Figure 4a,d). The broader shape of the triplet signal for **[(P3)_2_•(DABCO)_3_]^2−^** compared to **[(P3)_2_•(bipy)_3_]^2−^** reflects the stronger dipolar coupling in the DABCO complex due to the smaller electron–electron distance arising from a smaller ligand (distances between nitrogen atoms of 2.58 and 7.09 Å for DABCO and bipy, respectively). Initial simulations of an isolated triplet species using *pepper* ^[^ ^24^ ^]^ could provide approximate zero‐field splitting constants for the two systems (see Figure S4), but failed to accurately reproduce the signal shape and did not take into account the presence of both doublet and triplet signals. These issues were addressed by simulations of the time evolution of the spin density matrix presented below.

**Figure 4:**
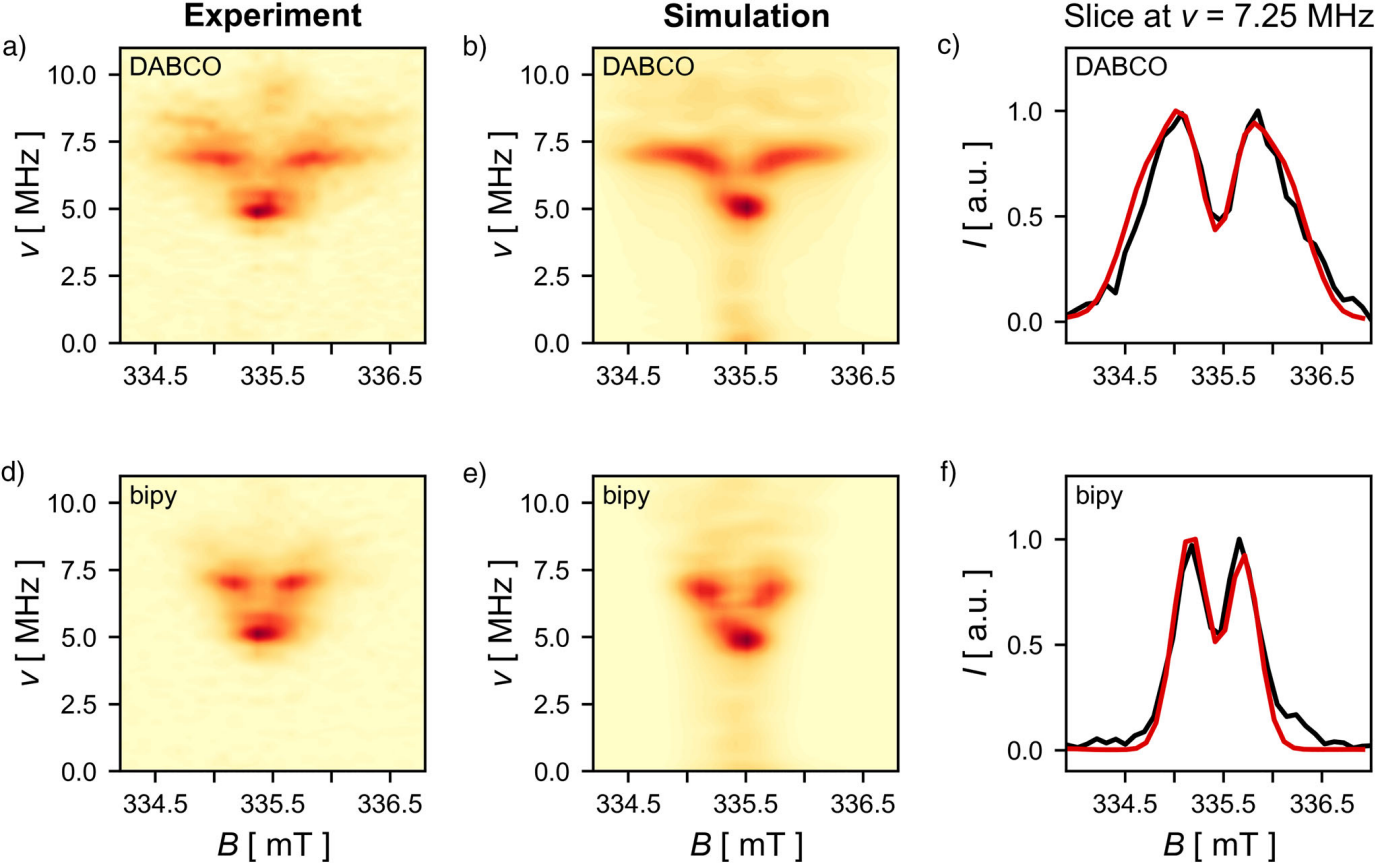
a,d) Experimental field‐dependent PEANUT spectra recorded at 80 K at X‐band frequencies in toluene for **[(P3)_2_•(bipy)_3_]^2−^** (bottom) and **[(P3)_2_•(DABCO)_3_]^2−^** (top) with a **P3** concentrations of 50 µM. b,e) Simulated spectra obtained from the Fourier transform of a spin dynamics simulation using dipolar coupling tensors calculated from DFT. c,f) Slice through the 2D PEANUT spectra at the triplet frequency for the experimental spectrum (black) and simulation (red).

The nutation spectra of weakly coupled electron spins can be simulated by calculating the time evolution of the density matrix under oscillating microwave irradiation. The simulations presented below are based on work by Ayabe et al., who showed that the nutation frequencies depend strongly on the magnitude of the coupling interactions, the difference between the *g*‐values of the electrons Δ*g* and the strength of the irradiating field ω_1_.^[^ ^25^ ^]^ Although they considered systems with an exchange and dipolar coupling term for a given orientation while varying the magnitude of *ω*_1_, here we consider the effect of changing the orientation of the dipolar interaction and thereby also changing the ratio between ω_1_ and the magnitude of the coupling interaction. The spin Hamiltonian considered here only includes the Zeeman interaction of the electrons and the secular and non‐secular terms of the dipolar coupling interaction between them. An expression for the dipolar coupling involving an orthorhombic dipolar coupling tensor was derived and depends on two angles *θ* and *ϕ* (as defined for polar coordinates, see Supporting Information) that describe the orientation of the dipolar tensor relative to the magnetic field. Details of the simulation method can be found in the Supporting Information.

The resulting 2D field‐dependent nutation simulations for **[(P3)_2_•(bipy)_3_]^2−^** and **[(P3)_2_•(DABCO)_3_]^2−^** (Figure 4b,e) bear a close resemblance to the experimental spectra, both in terms of the position of signals in the field and frequency directions and also the relative intensities. Furthermore, slices through the experimental spectra and simulations at the maximum of the triplet signal at ν ≈ 7.1 MHz show good agreement. Although these nutation simulations do not provide a simulation of the actual pulses in the PEANUT experiment, the numerical calculation of the nutation frequencies of all spin level transitions (within the field range) should provide a good description of the data collected by an ideal PEANUT experiment. Density functional theory (DFT) calculations were used to calculate the dipolar coupling tensor components *D* ^*ee*^ and *E* ^*ee*^ used in the simulations, as described below. The only parameters that were freely varied in the simulations were the line shape the spectrum was convoluted with and the difference between the *g*‐values of the electron spins Δ*g*. The latter did not greatly affect the spectral shape, but inclusion of a small difference (Δ*g* = 0.002) was required to correctly reproduce the relative positions of the nutation frequencies (see Figure S11). Note that the spin–spin interactions in these nutation simulations can be fully accounted for by the dipolar coupling and that no exchange coupling interaction was required to achieve good agreement between the experimental PEANUT spectra and the field‐dependent nutation simulations (see Figures S12 and S13 for further details).

For a better understanding of the contributions to the spectrum, the orientation dependences of the signals at *ν*_1_ and $\sqrt{2}\;\nu_{1}$ are shown for **[(P3)_2_•(DABCO)_3_]^2−^** in Figure 5a,b. The signals were integrated over the angle ϕ and are shown as functions of θ only. The pattern seen for the triplet signal resembles the familiar dipolar splitting pattern that also gives rise to the Pake powder pattern. However, the intensity decreases near the magic angle (θ = 54.7°) while the doublet‐like signal mainly originates from orientations close to this angle (see Figure 5c). For these orientations, it is not possible to selectively excite specific spin‐transitions as the secular part of the dipolar interaction and therefore the energy‐difference between the α_1_β_2_ and β_1_α_2_ spin‐levels approaches 0. The expression for the nutation frequency of a transition between two spin‐levels (Equation 1) only applies to selective excitations of a transition.^[^ ^21^ ^]^ Consequently, due to simultaneous excitation of multiple transitions, the nutation frequency of all transitions at the magic angle is given by ν_1_ instead. In addition, the pseudo‐secular term of the dipolar coupling that gives rise to the mixing between the spin levels α_1_β_2_ and β_1_α_2_ also approaches 0 near the magic angle and therefore no longer gives rise to a triplet spin wavefunction. A single chemical species consisting of two electron spins that are weakly coupled can therefore give rise to signals at multiple different nutation frequencies as has been observed before in other systems.^[^ ^25^, ^26^ ^]^ Such systems are therefore not well‐described by the expression in Equation 1. Note that due to the orthorhombicity of the dipolar coupling tensor in the ladder complexes, the dipolar coupling approaches 0 at different values of θ, depending on the value of ϕ. Therefore, the triplet frequency does not completely disappear near the magic angle (*θ* = 54.7°), and the doublet signal has two maxima above and below the magic angle.

**Figure 5:**
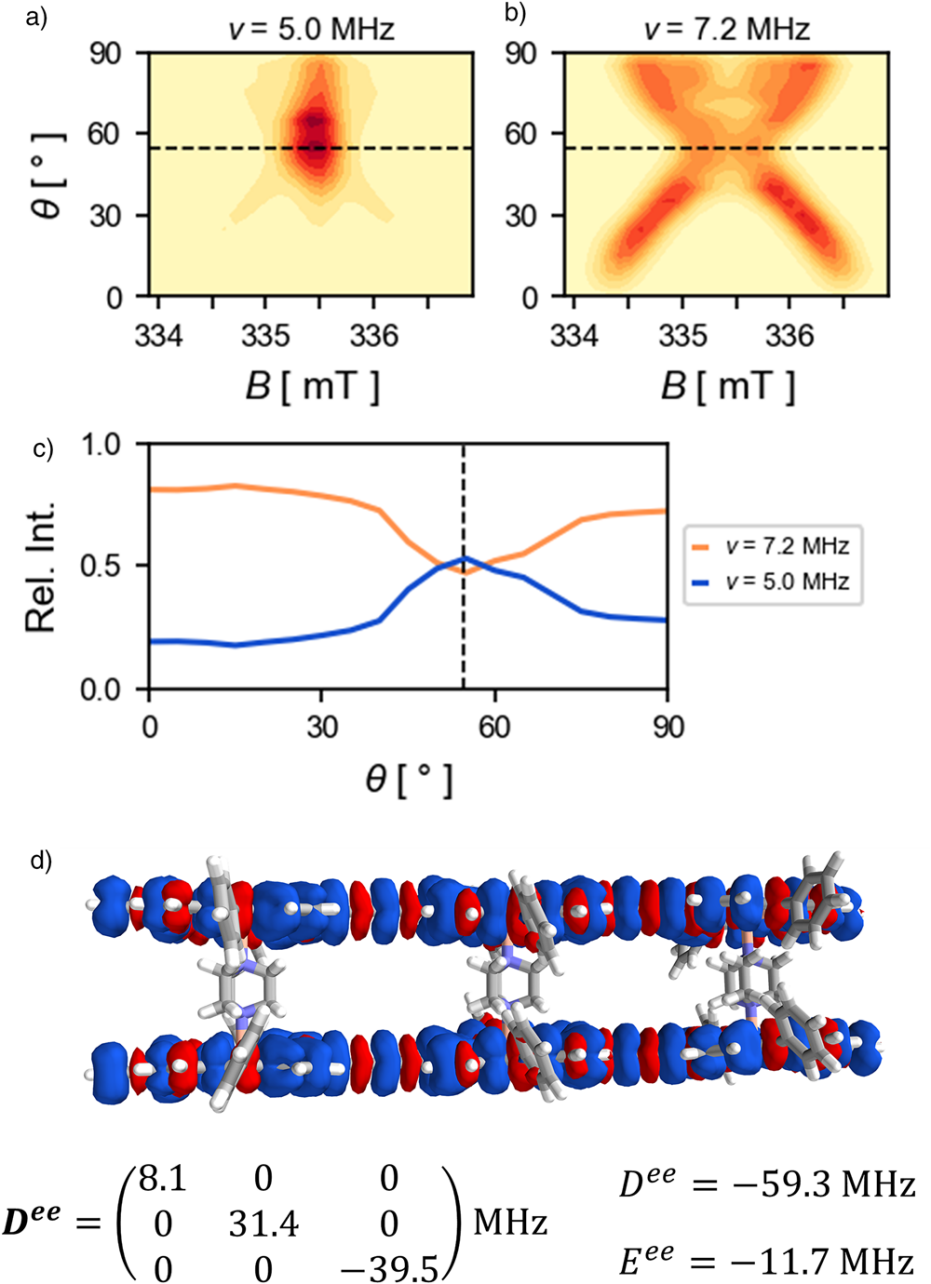
Orientation dependence of the signal at selected frequencies from the field‐dependent nutation simulations of **[(P3)_2_•(DABCO)_3_]^2−^** shown in Figure 4b. Slices were integrated over the angle ϕ and are shown as functions of θ for the signals appearing at the a) doublet (5 MHz) and b) triplet (7.2 MHz) frequencies. c) Relative signal intensities of the signals at doublet and triplet frequencies obtained from integrating along the field axis. The dotted lines indicate the magic angle (θ = 54.7). d) Spin density plot of **[(P3)_2_•(DABCO)_3_]^2−^** obtained from DFT calculations (lc‐*ω*PBE, *ω*=0.15) and dipolar coupling tensor calculated using the distributed point dipolar approximation.

The dipolar coupling parameters *D* ^*ee*^ and *E* ^*ee*^ used in the nutation simulations of the complexes **[(P3)_2_•(bipy)_3_]^2−^** and **[(P3)_2_•(DABCO)_3_]^2−^** were obtained from density functional theory calculations. The spin densities were calculated using the range‐separated functional lc‐ωPBE (ω = 0.15), which has previously been found to provide a good description of the monoanionic trimer **P3^•−^**.^[^ ^19^ ^]^ The spin densities are equally spread over the two porphyrin oligomers, as can be seen from the sum of the Mulliken atomic spin populations of 1.00 per **P3** unit in both complexes (see Figure 5 for DABCO complex). The SOMO (singly occupied molecular orbital) and SOMO‐1 are located on the two different porphyrin oligomers (see Figure S10), which means that the system can be viewed as having one unpaired electron per porphyrin chain with negligible amounts of spin density on the ligands (|ρ| ⩽ 0.002 per ligand). The dipolar coupling tensors were calculated using the distributed point dipolar approximation^[^ ^27^ ^]^ (see Supporting Information for details), and the resulting tensor for **[(P3)_2_•(DABCO)_3_]^2−^** is shown in Figure 5d. Due to the considerable delocalization of spin density over the π‐system of **P3**, the tensor shows appreciable deviations from axial symmetry (*E* ≠ 0). The dipolar coupling constants for the complex **[(P3)_2_•(bipy)_3_]^2−^** (*D* ^*ee*^ = −26.1 MHz, *E* ^*ee*^ = −4.3 MHz) are smaller than for **[(P3)_2_•(DABCO)_3_]^2−^** (*D* ^*ee*^ = −59.3 MHz, *E* ^*ee*^ = −11.7 MHz) as expected due to the larger distance between π surfaces. The dipolar coupling parameters obtained this way resulted in the simulated spectra shown in Figure 4; these are in good agreement with the experimental spectra for both complexes, thus supporting the ladder complexes as the origin of the observed triplet spectra.

This work explored a new class of supramolecular diradical formed by the self‐assembly of porphyrin oligomer radical anions. Although the direct aggregation of oligomer anions is difficult to interpret, the use of well‐established ladder formation enabled the controlled study of systems with two electron spins. The delocalization of the spin density across the respective π‐systems of the porphyrin oligomers results in an orthorhombic dipolar coupling tensor, and the resulting expressions for the secular and pseudo‐secular terms of the dipolar coupling interaction were derived. Due to the orientation dependence of these terms, the splitting of the energy levels approaches 0 for some orientations, which prevents a selective excitation of individual spin transitions. Consequently, field‐dependent PEANUT experiments result in signals at two different nutation frequencies with a ratio of $\sqrt{2}$ and simulations based on the time evolution of the density matrix showed that both signals can arise from the same spin system. The good agreement between the DFT‐calculated dipolar interaction tensors and the experimental spectra supports that the triplets originate from the charged species of a known supramolecular structure that is discrete and well‐defined. A range of similar supramolecular complexes has previously been studied in their neutral states^[^ ^23^, ^28^, ^29^, ^30^ ^]^ and introduction of charge carriers could give rise to similar spin systems as presented here. Although we demonstrate ladders of radical anions, similar structures could probably be assembled from radical cations. The ability to switch between different spin multiplicities may have potential applications in quantum information science, but there are still many challenges involved in addressing and integrating functional devices.^[^ ^31^ ^]^ This study represents a new strategy for the formation of triplet species arising from weak coupling interactions and might inspire the design of other such diradical systems with possible applications in dynamic nuclear polarization, spintronics, or supramolecular electronics.

## Conflict of Interests

The authors declare no conflict of interest.

## Supporting information

Supporting Information

## Data Availability

The data that support the findings of this study are available in the Supporting Information of this article.
