# Supplementary material for: Spin Interactions in Supramolecular Assemblies of Porphyrin Oligomer Radical Anions
Source: Angew Chem Int Ed Engl. 2025 Oct 7;64(47):e202518425. doi: 10.1002/anie.202518425 (PMC12624324; doi:10.1002/anie.202518425)
Supplement: Supplementary file 1 — Supporting Information [file ANIE-64-e202518425-s001.pdf]

Supporting Information

Spin Interactions in Supramolecular Assemblies of Porphyrin Oligomer Anions

Janko Hergenhahn,[a,b] Sebastian M. Kopp,[a,b] Henrik Gotfredsen,[a] Kan Tang,[c] Stephen Barlow,[c] Seth R. Marder,[c,d] Christiane R. Timmel*,[a,b] and Harry L. Anderson*,[a]

[a] Chemistry Research Laboratory, Department of Chemistry, University of Oxford, Oxford, OX1 3TA, UK

[b] Centre for Advanced Electron Spin Resonance, Department of Chemistry, University of Oxford, Oxford, OX1 3QR, UK

[c] Renewable and Sustainable Energy Institute, University of Colorado Boulder, Boulder, CO 80309, USA

[d] Departments of Chemical and Biological Engineering and of Chemistry, University of Colorado Boulder, Boulder, CO 80309, USA

Contents

1. Methods 2
1.1 Experimental methods 2
1.1.1 Sample preparation for EPR measurements 2
1.1.2 EPR measurements 2
1.1.1 UV-VIS-NIR measurements 3
1.2 Computational methods 3
1.2.1 DFT calculations 3
1.2.2 Nutation Simulations 4
2. Reduction CW EPR titration on **P3** 5
3. Comparison of reducing agents 6
4. Comparison of doublet spectra 7
5. Numerical triplet simulations 7
6. UV-VIS titrations 8
7. Pulsed EPR Relaxation Measurements 10
8. Derivation of dipolar interaction for orthorhombic dipolar tensor 11
9. DFT Calculations 12
10. Role of g-value offset and exchange coupling 13
References 15

1. Methods

1.1 Experimental methods

1.1.1 Sample preparation for EPR measurements

The butadiyne-linked porphyrin trimer **P3** was synthesized as previously reported.[1] All samples were prepared in spectroscopy-grade toluene that was filtered through dry alumina and purged of oxygen prior to use. Measurements on ladder complexes were done by first forming the 2:3 ladder complex by addition of 1.6 equivalents of the bidentate ligand (DABCO or bipy) to a 50 µM solution of **P3**. The successful formation of the neutral complex was verified by UV-VIS-NIR spectroscopy. Reductions were done under an inert nitrogen atmosphere inside a glove box (MBraun LabStar Glovebox) using $(N\text{-}DMBI)_2$ (dissolved in toluene) by addition of 1.1 equivalents of reducing agent (per porphyrin oligomer). While the reduction with $(N\text{-}DMBI)_2$ has been found to be slow in previous UV-VIS spectroscopy experiments,[2] the reduction process of the EPR samples appeared to happen over the course of just a few seconds as judged by the change of color of the solution, probably due to the higher concentration of the reducing agent and porphyrin solutions. Samples were prepared directly inside a 3.8 mm X-band EPR tube. The EPR tube was closed with a tight-fitting cap and shock-frozen in liquid N_2 after removal from the glove box.

1.1.2 EPR measurements

Pulsed EPR measurements were carried out at 80 K at X-band frequencies using a Bruker E580 spectrometer. Hahn echo detected field sweeps were recorded with pulse lengths of $t_{\pi/2}$ = 16 ns and t_π = 32 ns and a delay of τ = 300 ns.

Field-dependent PEANUT experiments were recorded using the pulse sequence $\frac{\pi}{2} - \tau - p_x - p_{-x} - \tau - echo$ with $t_{\pi/2}$ = 50 ns and τ = 200 ns.[3] The combined pulse length of p_x and p_{-x} was 3072 ns. This was long enough to prevent overlapping of the rotary echo in the center and the nutation decays at the start and end of the time traces, which was confirmed by measuring the PEANUT trace at a single field point by varying p_x from 24 ns to 3048 ns (and p_{-x} from 3048 ns to 24 ns). The actual measurements only recorded the central region to save on measurement time and p_x was only varied from 1024 ns to 2048 ns (and correspondingly p_{-x} from 2048 ns to 1024 ns) with a time step of 4 ns (256 steps). The field-dependent PEANUT spectrum was recorded with a field resolution of 0.1 mT (about 30 to 40 steps).

The field-dependent PEANUT signal was processed by first applying a baseline correction by subtracting a linear baseline along the time axis for each field point. The signal was then apodized with a Gaussian function (centered at the center of the time trace) and zero-filled to 2048 data points along the time axis. Finally, the Fourier transform was calculated along each time trace and the absolute value was taken to obtain the final 2D-field-frequency spectrum.

Spin-spin relaxation times (T_m) were measured using 2-pulse echo decay experiments with the pulse sequence $\frac{\pi}{2} - \tau - \pi - \tau - echo$ with pulse lengths of $t_{\pi/2}$ = 16 ns and t_π = 32 ns. The delay time τ was varied between 180 ns and about 16000 ns in steps of 16 ns. Spin-lattice relaxation times (T_1) were measured using inversion recovery experiments with the pulse sequence $\pi - T - \pi/2 - \tau - \pi - \tau - echo$ with pulse lengths of $t_{\pi/2}$ = 16 ns and t_π = 32 ns. The waiting time T was varied between 0.3 µs and 40,000 µs or 80,000 µs (depending on the system) in steps of 100 µs.

1.1.1 UV-VIS-NIR measurements

UV-VIS-NIR spectra were recorded in a quartz cuvette on a Cary60 UV-Vis-NIR spectrophotometer. UV-VIS titrations were done at room temperature in spectroscopy-grade toluene.

A binding titration between a reduced porphyrin monomer $\mathbf{P1}^{\bullet-}$ and pyridine was carried out in a Quartz UV-VIS flat-cell with a path length of 1 mm and a J. Young cap. The porphyrin monomer was reduced prior to the titration with an excess of reducing agent, $(N\text{-}DMBI)_2$ dissolved in toluene, inside a glove box (MBraun LabStar Glovebox). Each addition of pyridine was also done inside the glove box to maintain an oxygen-free environment. To ensure that the concentration of the porphyrin remained constant during the titration, the titrant solution contained the same concentration of porphyrin as the sample inside the cell. The binding constant K_a was estimated by fitting to a 1:1 binding isotherm given by:

$$\frac{A-A_{initial}}{A_{\infty}-A_{initial}} = \frac{(K_a([L]+[P]_0)+1)-\sqrt{(K_a([L]+[P]_0)+1)^2-4K_a^2[P]_0[L]}}{2K_a[P]_0} \quad \text{(S1)}$$

where A is the absorption at a certain wavelength (and $A_{initial}$ and A_{∞} are the starting absorption and asymptotic final absorption respectively), $[L]$ is the total concentration of the ligand and $[P]_0$ is the total concentration of the porphyrin (which is kept constant during the titration). This analysis gave a binding constant of K_a = (4.8 ± 0.1) × 10^3 M^{-1} for coordination of pyridine to the zinc center of $\mathbf{P1}^{\bullet-}$ N-$DMBI^+$ in toluene at 298 K (see Section 6 below). This binding constant is approximately 1/5 the binding strength found for the neutral porphyrin monomer under these conditions ((2.7 ± 0.2) × 10^4 M^{-1}).

The UV-VIS-NIR titrations for the formation of the neutral ladder complexes were done in a standard 10 mm path quartz cuvette. The titrant was added directly to the porphyrin solution inside the cuvette under normal atmospheric conditions. After each addition, the sample was thoroughly mixed by a magnetic stir bar inside the cuvette. See resulting titration curves in Section 6 below.

1.2 Computational methods

1.2.1 DFT calculations

Structures of the porphyrin oligomers were optimized at the PBE0+D3(BJ)/def2-SVP(H, C, N)/def2-TZVP(Zn) level of theory using Gaussian 16 with a tight convergence threshold. The charge of the system was set to 2– and the overall multiplicity to S = 1. Calculations of the spin densities were then carried out on the optimized structures in ORCA 5.0 using the lc-ωPBE functional with ω = 0.15.[4]

The dipolar coupling tensor was obtained from the calculated spin densities using the distributed point dipolar approximation,[5] which evaluates the contributions to the dipolar coupling between each atom over which electron 1 is distributed with each atom over which electron 2 is distributed:

$$\boldsymbol{D}^{ee} = -\frac{\mu_0}{4\pi\hbar} g_e^2 \mu_B^2 \sum_{i,j} \rho_i \rho_j \frac{3\,\hat{\boldsymbol{n}}_{ij} \otimes \hat{\boldsymbol{n}}_{ij} - \mathbf{1}}{r_{ij}^3} \quad \text{(S2)}$$

Where μ_0 is the magnetic constant, g_e is the electron g-factor, μ_B is the Bohr magneton, r_{ij} and $\hat{\boldsymbol{n}}_{ij}$ are the distance and direction unit vector between atoms i and j and ρ_i and ρ_j are the Mulliken spin populations on atoms i and j. The expression above gives the dipolar coupling tensor in units of angular frequency.

1.2.2 Nutation Simulations

Numerical simulations of the nutation experiments were performed by considering the time evolution of the spin density matrix in the uncoupled basis ($|\alpha_1\alpha_2\rangle, |\alpha_1\beta_2\rangle, |\beta_1\alpha_2\rangle, |\beta_1\beta_2\rangle$). The Hamiltonian contained the Zeeman interactions of the two electrons as well as the secular and pseudo-secular dipolar coupling terms. This Hamiltonian was transformed into the rotating frame so that the irradiating field can be described by a time-independent magnetic field in the xy-plane.[6]

The full procedure for the simulations is given below:

1. Calculating the base Hamiltonian for a given orientation (θ and ϕ) and field B_0:

$$\begin{aligned}\widehat{H}_0 = \ & \frac{g_1\mu_B B_0}{\hbar}\hat{S}_{1z} + \frac{g_2\mu_B B_0}{\hbar}\hat{S}_{2z} \\ & + \left[\frac{D^{ee}}{3}(3\cos^2\theta - 1) + E^{ee}\sin^2\theta\cos 2\phi\right]\hat{S}_{1z}\hat{S}_{2z} \\ & - \frac{1}{4}\left[\frac{D^{ee}}{3}(3\cos^2\theta - 1) + E^{ee}\sin^2\theta\cos 2\phi\right](\hat{S}_{1+}\hat{S}_{2-} + \hat{S}_{1-}\hat{S}_{2+})\end{aligned} \tag{S3}$$

2. Transforming the Hamiltonian into the rotating frame:

$$\widehat{H}_{0,rot} = \widehat{H}_0 - \omega_{mw}(\hat{S}_{1z} + \hat{S}_{2z}) \tag{S4}$$

3. Calculating the full Hamiltonian by including the irradiation field:

$$\widehat{H}_{1,rot} = \omega_1(\hat{S}_{1x} + \hat{S}_{2x}) \tag{S5}$$

$$\widehat{H}_{rot} = \widehat{H}_{0,rot} + \widehat{H}_{1,rot} \tag{S6}$$

4. Setting the density matrix at $t = 0$ to $\rho_0 = -(\hat{S}_{1z} + \hat{S}_{2z})$
5. Evaluating the evolution of the density matrix for each time point using:

$$\rho(t) = \widehat{U}(t)\rho_0\widehat{U}^{\dagger}(t) \tag{S7}$$

$$\widehat{U}(t) = e^{-i\widehat{H}_{rot}t} \tag{S8}$$

6. Calculating the *xy*-magnetization $M_{xy}(t) = \langle\hat{S}_x(t)\rangle + i\langle\hat{S}_y(t)\rangle$ by evaluating:

$$\langle\hat{S}_q(t)\rangle = tr\left[(\hat{S}_{1q} + \hat{S}_{2q})\rho(t)\right] \tag{S9}$$

for *q* = *x,y* and background correcting them each by subtracting the time average of the respective$\langle\hat{S}_q(t)\rangle$.

7. This procedure is repeated for every orientation for a given field and then for each field point.

All simulations used a time step of 8 ns and 256 steps with a total time vector length of 2048 ns. The simulations used between 30 and 40 field points and orientations (θ and ϕ) were generated by using the *sphgrid* function provided by *EasySpin* using D_{2h} symmetry. The resulting time traces were then processed similarly to how the experimental PEANUT time traces were processed. Approximately the first 10% of each time trace was removed to obtain a steady state of the nutation. The time traces were then apodized using a Gaussian function (centered at 0) and zero-filled to 2048 points. Subsequently, the Fourier transform was taken to obtain the frequency spectrum and the final spectrum was obtained by convolution with a Gaussian line shape with a width of 0.2 MHz.

2. Reduction CW EPR titration on P3

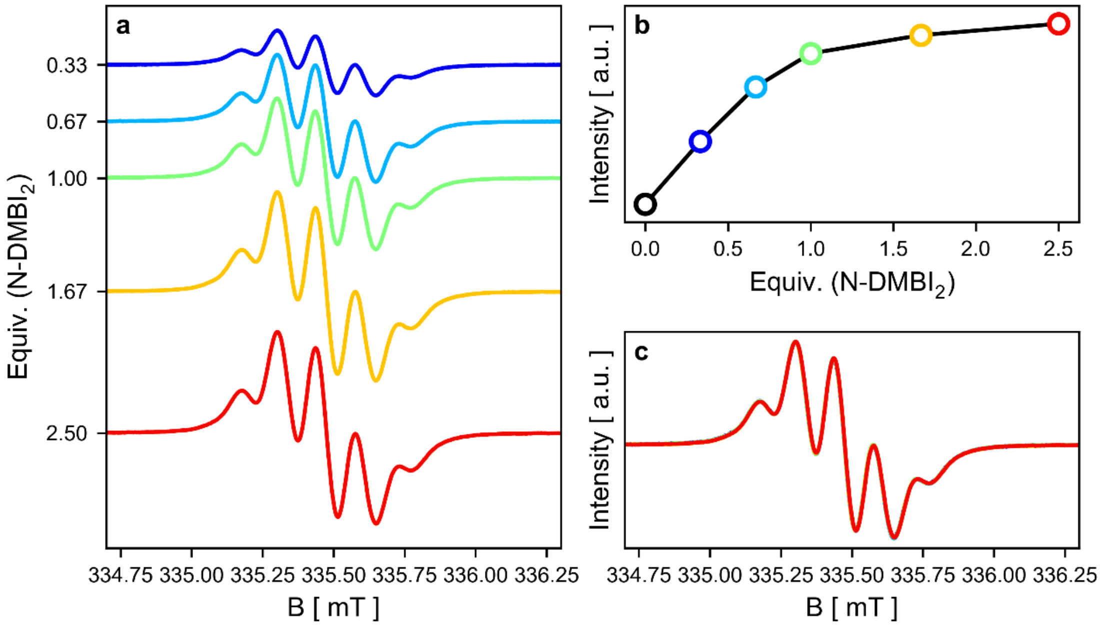

Figure S1. a) CW-EPR spectra of **P3** recorded in THF (200 µM) with 0.1 M TBAP at room temperature and X-band frequency (9.40 GHz) with varying amounts of reducing agent (N-DMBI)$_2$. b) Signal intensities for different points during the titration determined from the double integral of the signal after background correction. c) Overlayed normalized spectra from plot a) to show that the signal shape did not change over the course of the titration.

3. Comparison of reducing agents

We have tested what effect different reducing agents have on the shape of the room temperature CW-EPR spectrum porphyrin radical anions using the previously investigated monomer **P1** as a test system. In an earlier study, we have found that reduction of **P1** to **P1**$^{\bullet-}$ using decamethylcobaltocene ($CoCp^*_2$) results in a distortion of the CW-EPR spectrum likely due to the formation of a tight ion pair complex with the positively-charged counter ion of the reducing agent (chemical structure of **P1**$^{\bullet-}$ shown in Figure S7). It was possible to prevent this effect by addition of an inert electrolyte such as tetrabutylammonium hexafluorophosphate (TBAP) which resulted in the expected CW-EPR spectrum of the free **P1**$^{\bullet-}$ ion consistent with DFT-calculations.[7]

Here, we show that the use of different, bulkier reducing agents such as $(N\text{-}DMBI)_2$ and $[RuCp^*(mes)]_2$ (see Figure S2) also results in CW-EPR spectra of the free **P1**$^{\bullet-}$ ion. This indicates that these reucing agent do not lead to the aggregation seen with $CoCp^*_2$, even when using a less polar solvent such as toluene. The absence of ion pair complexes and the use of toluene are crucial for the study of the reduced ladder complexes such as **$[(P3)_2{\cdot}(DABCO)_3]^{2-}$** and **$[(P3)_2{\cdot}(bipy)_3]^{2-}$** as the ladder complex formation is not feasible with the presence of THF as a competing ligand. On top of these precautions, it should be noted that the formation of a tight-ion pair complex between a radical porphyrin monoanion and a positively charged reducing agent counter ion becomes less likely for larger porphyrin oligomers due to the decreasing charge density from delocalization of the additional electron over the conjugated π-system.

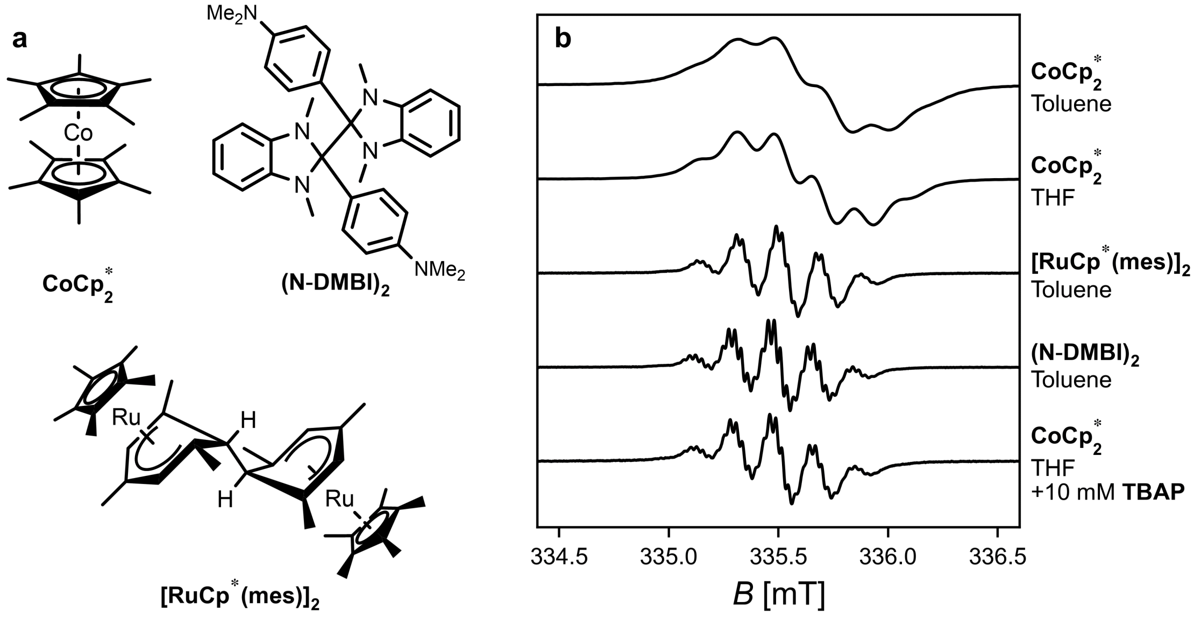

Figure S2. CW-EPR spectra of **P1**$^{\bullet-}$ recorded with different solvent conditions and reducing agents (shown on the left) at 298 K at X-band frequencies. All samples were measured with a concentration of 200 µM.

4. Comparison of doublet spectra

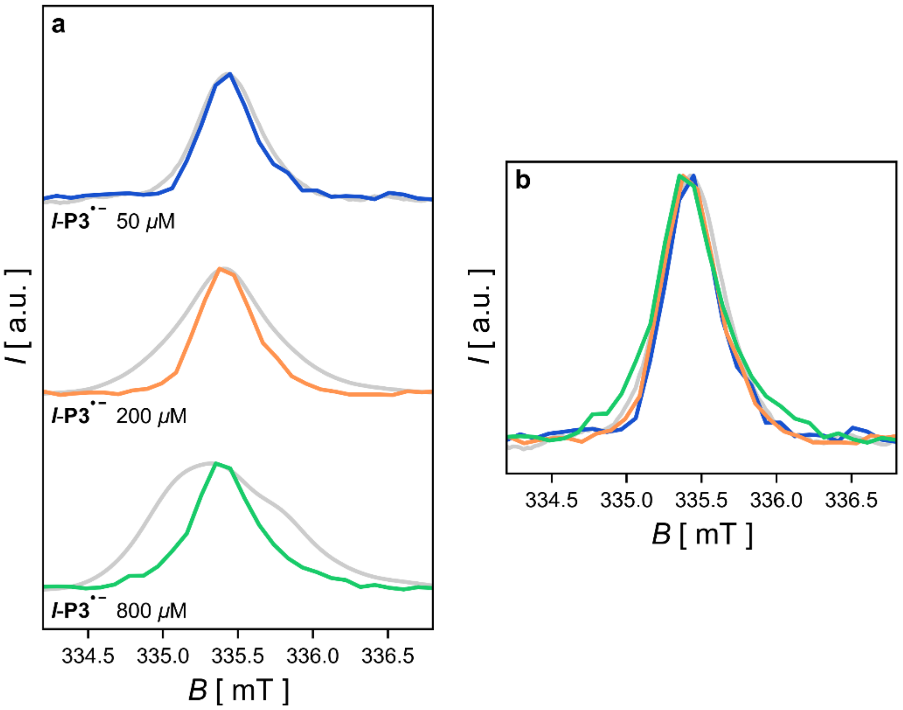

Figure S3. a) Comparison of slices through the PEANUT spectra at the frequency corresponding to S = 1/2 for solutions of $\mathbf{P3^{\bullet-}}$ in toluene recorded at different concentrations at 80 K at X-band frequencies. The respective echo-detected field sweep spectra are shown in gray for comparison. b) The same spectra as shown in (a) but overlayed on top of each other. Echo-detected field sweep spectrum of the 50 μM solution is shown in gray for reference.

5. Numerical triplet simulations

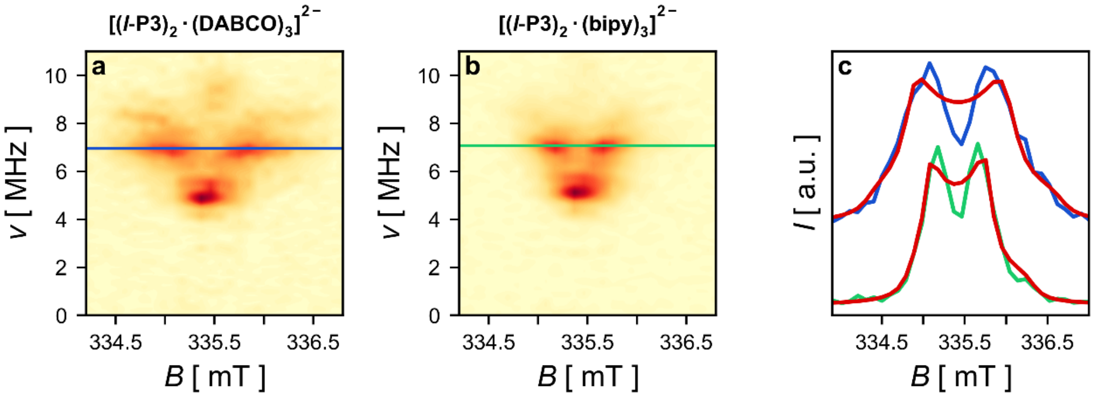

Figure S4. PEANUT field sweep spectra of a) $\mathbf{[(P3)_2{\cdot}(DABCO)_3]^{2-}}$ and b) $\mathbf{[(P3)_2{\cdot}(bipy)_3]^{2-}}$ recorded at 80 K at X-band frequencies in toluene. c) Slices through PEANUT field sweep spectra at the triplet frequency as indicated in a) and b) for DABCO complex (blue) and bipy complex (green) with simulations (red). The simulations are the best-fit obtained from simulations of a triplet species with orthorhombic D-tensor using the *pepper* function in *EasySpin*. These simulations cannot fully reproduce the dip in the center of the spectrum but give an estimate of the zero-field splitting tensor by simulating the width of the spectra (D = 32.4 MHz and E = 6.7 MHz for $\mathbf{[(P3)_2{\cdot}(DABCO)_3]^{2-}}$; D = 20.9 MHz and E =9.3 MHz for $\mathbf{[(P3)_2{\cdot}(bipy)_3]^{2-}}$).

6. UV-VIS titrations

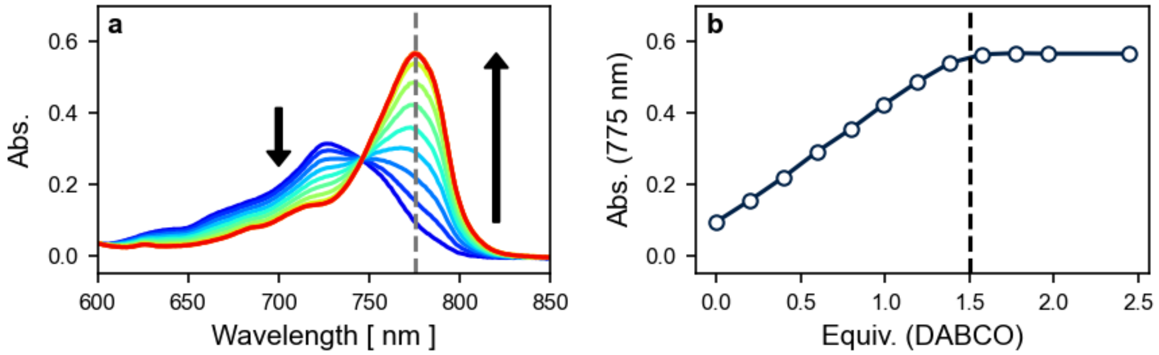

Figure S5. a) Q-band region for a VIS-NIR titration of **P3** with DABCO (298 K, in toluene, [**P3**] = 2.0 µM). b) Binding curve for the titration in (a) at the maximum absorption wavelength of the ladder complex (775 nm). Dotted line shows a stoichiometry of 2:3 (**P3**:DABCO).

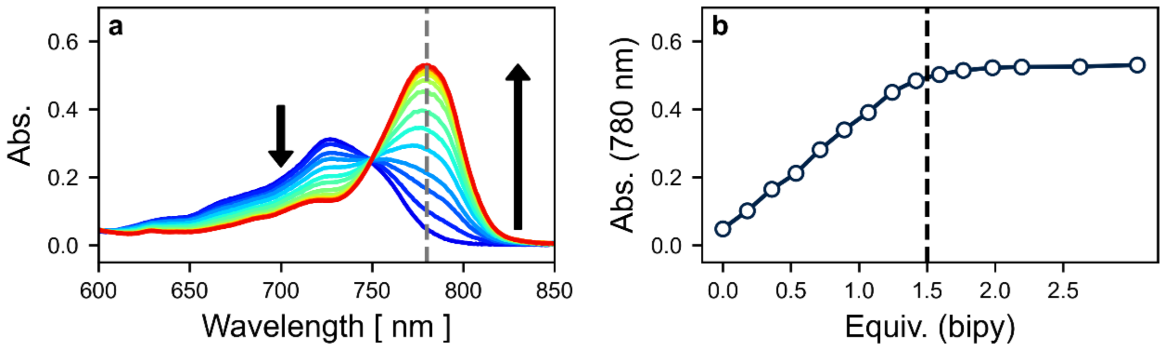

Figure S6. a) Q-band region for a VIS-NIR titration of **P3** with bipy (298 K, in toluene, [**P3**] = 2.0 µM). b) Binding curve for the titration in (a) at the maximum absorption wavelength of the ladder complex (780 nm). Dotted line shows a stoichiometry of 2:3 (**P3**:bipy).

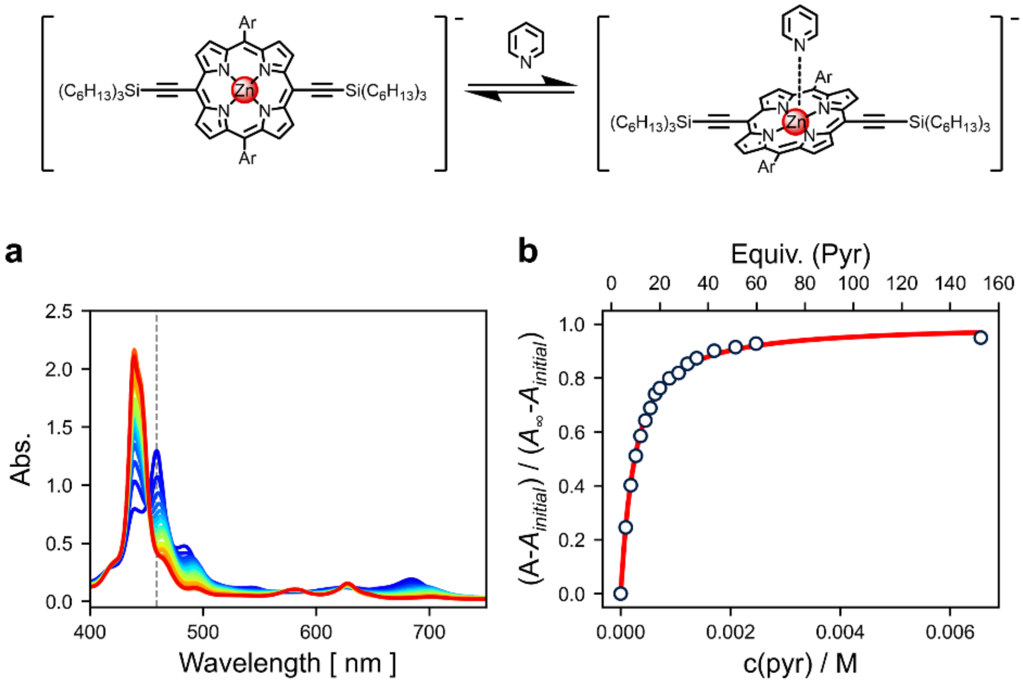

Figure S7. Binding of pyridine to a porphyrin monomer anion **P1**$^{\bullet-}$ (Ar= 3,5-bis(trihexylsilyl)phenyl (THS)). a) UV-VIS titration of **P1**$^{\bullet-}$ N-DMBI^{+} with pyridine (298 K, in toluene, [**P1**] = 92.0 µM). b) binding curve for the wavelength indicated in plot (a) (459 nm). Fitting a 1:1 binding isotherm resulted in a binding constant of (4.8 ± 0.1) × 10^{3} M^{-1}. This is approximately 1/5 the binding strength found for the neutral system ((2.7 ± 0.2) × 10^{4} M^{-1}).[8]

7. Pulsed EPR Relaxation Measurements

Relaxation times were measured for the free radical anion **P3$^{•–}$** and the ladder complex **[(P3)$_2$·(DABCO)$_3$]$^{2–}$**. The complex formation results in a significant 70% decrease in the T_1 relaxation time and a more modest decrease in the T_m relaxation time by about 25 %. The relaxation times of the **[(P3)$_2$·(DABCO)$_3$]$^{2–}$** complex were measured in different field positions but no significant variation in relaxation times was observed across the spectral shape.

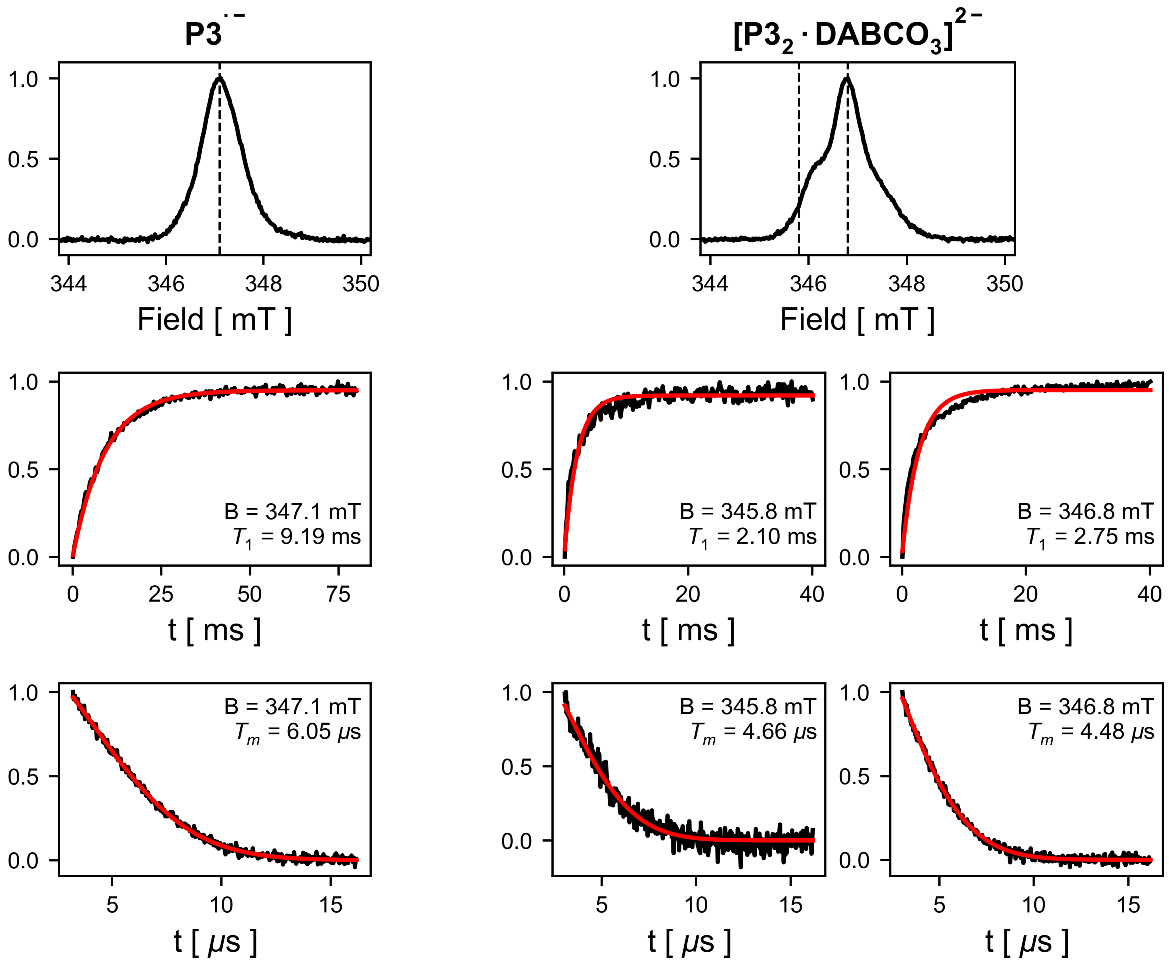

Figure S8. Field sweep EPR spectra (top), inversion recovery (middle) and echo decay (bottom) experiments of free **P3$^{•–}$** and **[(P3)$_2$·(DABCO)$_3$]$^{2–}$** recorded at 80 K at X-band frequencies in toluene with a **P3** concentration of 50 µM. Field positions at which the relaxation measurements were done are indicated by dashed lines in the field sweep spectra.

8. Derivation of dipolar interaction for orthorhombic dipolar tensor

The dipolar coupling tensor for an orthorhombic dipolar interaction (e.g. due to delocalization of the electron spin) in its eigenframe is given by:

$$\boldsymbol{D_0^{ee}} = \begin{pmatrix} -\frac{1}{3}D^{ee} + E^{ee} & 0 & 0 \\ 0 & -\frac{1}{3}D^{ee} - E^{ee} & 0 \\ 0 & 0 & \frac{2}{3}D^{ee} \end{pmatrix}$$

To transform the tensor into an arbitrary frame, one can apply three (Euler) rotations. In the *zyz* convention, the first rotation is around the *z*-axis by an angle ϕ, followed a rotation around the new *y*-axis by an angle θ and finally followed by a rotation around the new *z*-axis by an angle χ. The secular and pseudo-secular terms of the Hamiltonian are invariant to the third rotation (e.g. the final rotation around the *z*-axis leaves the $\hat{S}_{1z}\hat{S}_{2z}$ component unchanged). Therefore, only the first two rotations need to be considered. The first two rotations can be described by the rotation matrices $\boldsymbol{R_1}$ and $\boldsymbol{R_2}$:

$$\boldsymbol{R_1} = \begin{pmatrix} \cos\theta & 0 & \sin\theta \\ 0 & 1 & 0 \\ -\sin\theta & 0 & \cos\theta \end{pmatrix} \quad \boldsymbol{R_2} = \begin{pmatrix} \cos\theta & \sin\theta & 0 \\ -\sin\theta & \cos\theta & 0 \\ 0 & 0 & 1 \end{pmatrix}$$

The dipolar coupling tensor can be transformed from its eigenframe ($\boldsymbol{D_0^{ee}}$) to an arbitrary frame ($\boldsymbol{D^{ee}}$) and split into two terms that depending only on *D* or *E*:

$$\boldsymbol{D^{ee}} = \boldsymbol{R_1 R_2 D_0^{ee} R_2^T R_1^T} = \boldsymbol{D_D^{ee}} + \boldsymbol{D_E^{ee}}$$

$$\boldsymbol{D_D^{ee}} = \frac{D^{ee}}{3}\begin{pmatrix} 3\sin^2\theta - 1 & 0 & 3\sin\theta\cos\theta \\ 0 & -1 & 0 \\ 3\sin\theta\cos\theta & 0 & 3\cos^2\theta - 1 \end{pmatrix}$$

$$\boldsymbol{D_E^{ee}} = E^{ee}\begin{pmatrix} \cos^2\theta\cos 2\phi & \cos\theta\sin 2\phi & -\cos\theta\sin\theta\sin 2\phi \\ \cos\theta\sin 2\phi & -\cos 2\phi & -\sin\theta\sin 2\phi \\ -\cos\theta\sin\theta\sin 2\phi & -\sin\theta\sin 2\phi & \sin^2\theta\cos 2\phi \end{pmatrix}$$

The secular and pseudo-secular terms of the contributions of the dipolar interaction depend on the operators $\hat{S}_{1z}\hat{S}_{2z}$ and $\left(\hat{S}_{1+}\hat{S}_{2-} + \hat{S}_{1-}\hat{S}_{2+}\right)$ respectively.

By expanding the expression

$$\hat{H} = \boldsymbol{\hat{S}^T D^{ee} \hat{S}}$$

and grouping together the relevant terms for $\hat{S}_{1z}\hat{S}_{2z}$ and $\left(\hat{S}_{1+}\hat{S}_{2-} + \hat{S}_{1-}\hat{S}_{2+}\right)$, one obtains:

$$\hat{A} = \left[\frac{D^{ee}}{3}(3\cos^2\theta - 1) + E^{ee}\sin^2\theta\cos 2\phi\right]\hat{S}_{1z}\hat{S}_{2z}$$

$$\hat{B} = -\frac{1}{4}\left[\frac{D^{ee}}{3}(3\cos^2\theta - 1) + E^{ee}\sin^2\theta\cos 2\phi\right](\hat{S}_{1+}\hat{S}_{2-} + \hat{S}_{1-}\hat{S}_{2+})$$

These expressions are equivalent to $\hat{A}$ and $\hat{B}$ of the “dipolar alphabet” if $E^{ee} = 0$ and $D^{ee} = -3\omega_{dd}$.

9. DFT Calculations

$[(P3)_2•(DABCO)_3]^{2}$

$$\boldsymbol{D}^{ee} = \begin{pmatrix} 8.1 & 0 & 0 \\ 0 & 31.4 & 0 \\ 0 & 0 & -39.5 \end{pmatrix} \text{MHz}$$

$$D^{ee} = -59.3 \text{ MHz}$$

$$E^{ee} = -11.7 \text{ MHz}$$

$[(P3)_2•(bipy)_3]^{2-}$

$$\boldsymbol{D}^{ee} = \begin{pmatrix} 4.4 & 0 & 0 \\ 0 & 13.0 & 0 \\ 0 & 0 & -17.4 \end{pmatrix} \text{MHz}$$

$$D^{ee} = -26.1 \text{ MHz}$$

$$E^{ee} = -4.3 \text{ MHz}$$

Figure S9 Dipolar coupling tensors $\boldsymbol{D}^{ee}$ of **$[(P3)_2•(DABCO)_3]^{2-}$** and **$[(P3)_2•(bipy)_3]^{2-}$** (in frequency units) after transformation into their respective eigenframes. The principal components D^{ee} and E^{ee} are given below the tensors.

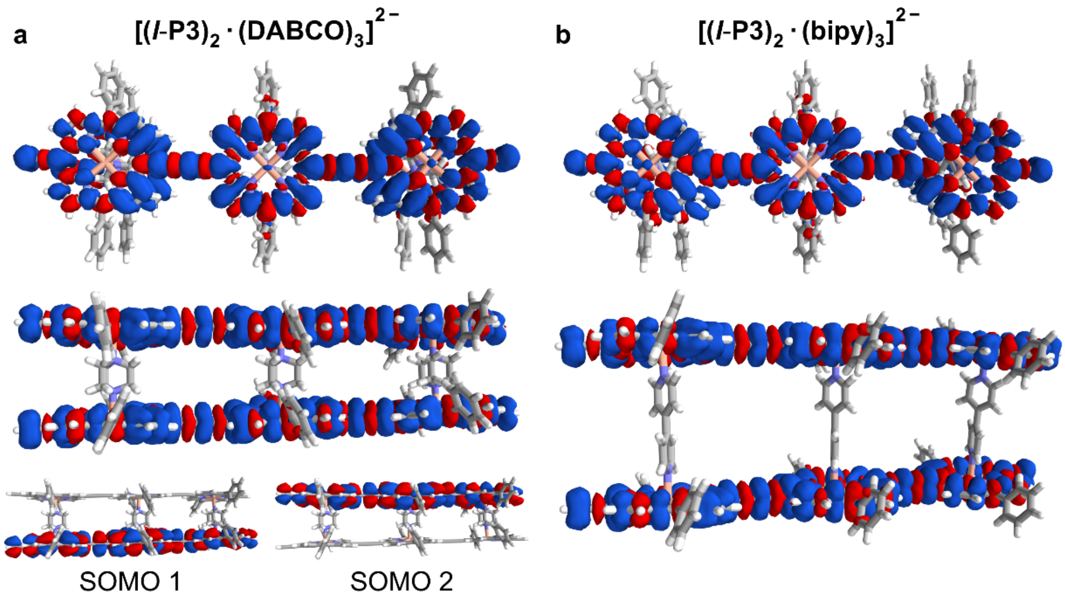

Figure S10. Spin density plots of a) **$[(P3)_2•(DABCO)_3]^{2-}$** and b) **$[(P3)_2•(bipy)_3]^{2-}$** from top and side. In addition, orbital plots for the pair of near-degenerate singly occupied molecular orbitals (SOMO) in **$[(P3)_2•(DABCO)_3]^{2-}$** are shown (bottom left). Structures were optimized using PBE0+D3BJ/def2-SVP(H,C,N)+def2-TZVP(Zn) and spin densities calculated with lc-ωPBE(ω=0.15)+D3BJ/EPR-II using ORCA 5.0.4.[9]

10. Role of g-value offset and exchange coupling

We explored the effect of the difference between the g-values of the electron spins (Δg) and the exchange coupling constant (J) on the field-dependent nutation simulations; this is shown exemplarily on the **[(P3)$_2$•(DABCO)$_3$]$^{2-}$** dianion complex below.

No exchange coupling or Δg was required to qualitatively simulate the field-dependent nutation simulations (Figure S11, first column from left). However, in the absence of a g-offset there is a small shift in the nutation frequency of the doublet-like signal from 5 MHz (ν_1) to about 5.4 MHz. Even very small values of Δg result in the doublet-like signal to shift towards 5 MHz but further increases in Δg result in a distorted shape of the triplet-like signal (see Figure S11). In fact, a small frequency shift of the doublet-like signal was also observed in the experimental PEANUT spectrum which resulted in a ratio of 1.38 between the two observed frequencies (instead of the expected $\sqrt{2}$ = 1.41) and repeated measurements resulted in frequency ratios between 1.34 and 1.40. This supports a very small magnitude of Δg resulting from the parallel conformation of the **P3$^{\bullet-}$** in **[(P3)$_2$•(DABCO)$_3$]$^{2-}$** and consequently aligned g-tensors.

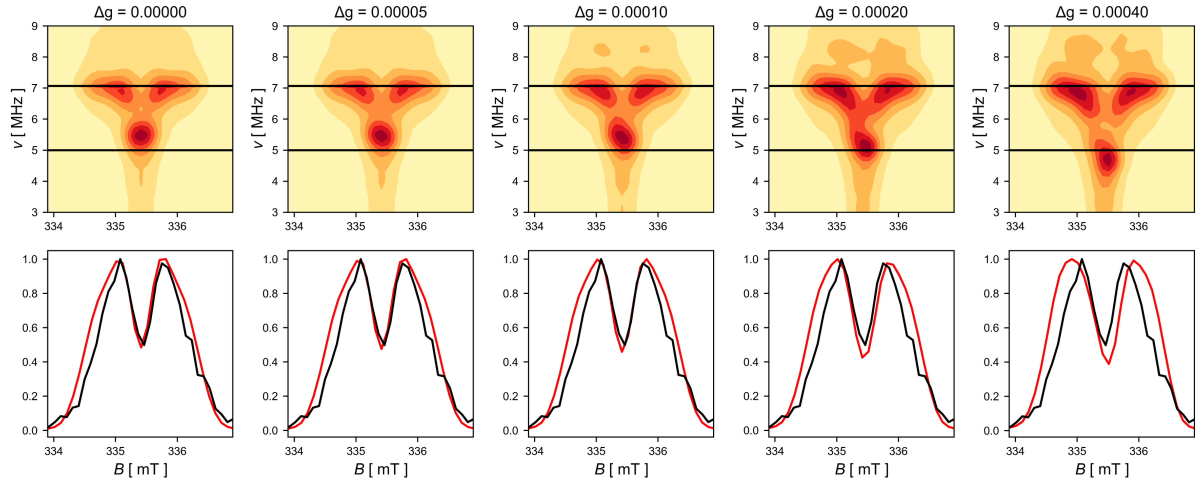

Figure S11. Field-dependent nutation simulations of **[(P3)$_2$•(DABCO)$_3$]$^{2-}$** with varying Δg (top row) with black lines indicating the ν_1 frequency (5 MHz) and $\sqrt{2}\nu_1$ (7.07 MHz). Slices through the field-dependent nutation simulation at the triplet frequency $\sqrt{2}\nu_1$ (bottom row) are shown in red and the experimental PEANUT spectrum in black (see main text for details). Simulations only included Zeeman interaction and dipolar coupling as described in Section 1.2.2.

The effect of exchange on the field-dependent nutation simulations was tested by including the exchange coupling term $H_{ex} = J\,\hat{\boldsymbol{S}}_1 \cdot \hat{\boldsymbol{S}}_2$ in the spin Hamiltonian shown in equation E3 in Section 1.2. Figures S12 and S13 show the different effects the exchange coupling has on the simulations in the absence and in the presence of a g-offset Δg for values of J between 0 and 100 MHz. For the case where $\Delta g = 0$, the exchange coupling has no visible effect on the simulations. In contrast, for the system with $\Delta g = 0.0002$ the exchange coupling term initially results in a distorted spectral shape and for larger values of J an additional nutation frequency appears at an intermediate frequency. This is not consistent with the experimentally observed PEANUT spectrum which indicates that only a small amount of exchange coupling is present in these systems or the very small difference in the g-values between the two spin centres makes it difficult to observe and masks its presence.

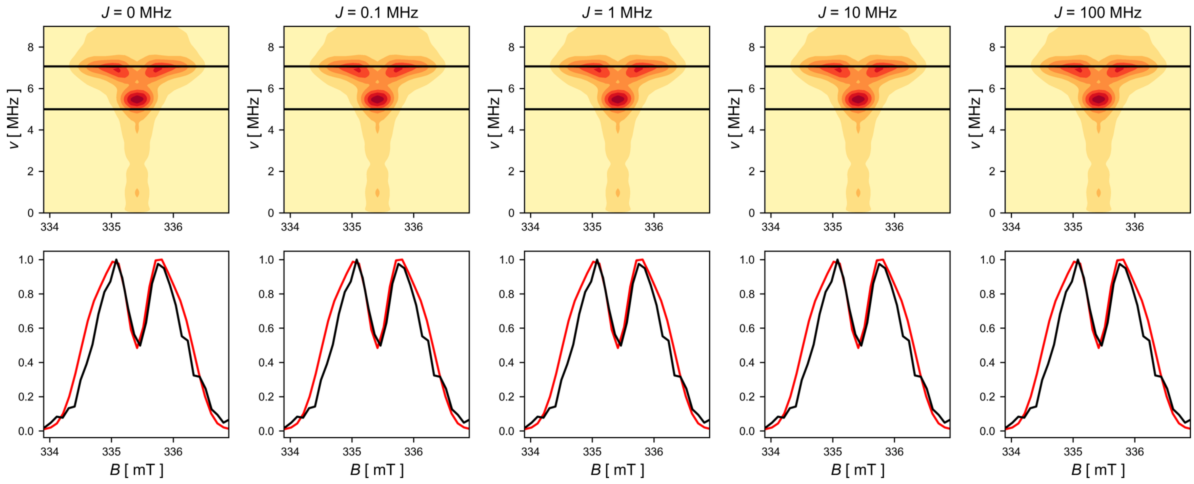

Figure S12. Field-dependent nutation simulations of **[(P3)₂•(DABCO)₃]²⁻** with varying J for $\Delta g = 0$ (top row) with black lines indicating the ν_1 frequency (5 MHz) and $\sqrt{2}\nu_1$ (7.07 MHz). Slices through the field-dependent nutation simulation at the triplet frequency $\sqrt{2}\nu_1$ (bottom row) are shown in red and the experimental PEANUT spectrum in black (see main text for details).

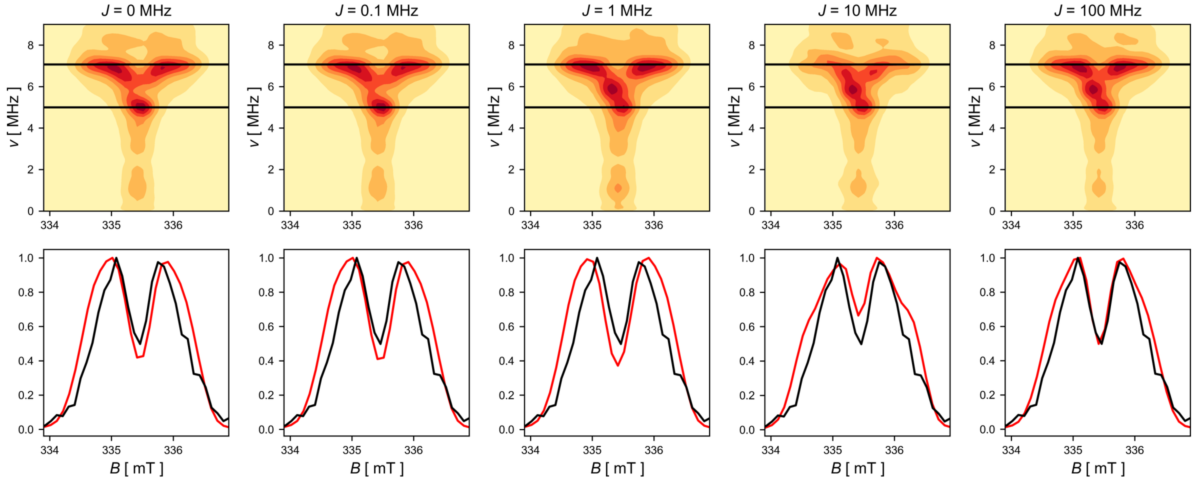

Figure S13. Field-dependent nutation simulations of **[(P3)₂•(DABCO)₃]²⁻** with varying J for $\Delta g = 0.002$ (top row) with black lines indicating the ν_1 frequency (5 MHz) and $\sqrt{2}\nu_1$ (7.07 MHz). Slices through the field-dependent nutation simulation at the triplet frequency $\sqrt{2}\nu_1$ (bottom row) are shown in red and the experimental PEANUT spectrum in black (see main text for details).

In order to estimate the magnitude of the exchange coupling, we attempted broken-symmetry density functional theory (BS-DFT). The ladder complex dianions were optimized with PBE0+D3BJ/def2-SVP(H,C,N)+def2-TZVP(Zn) with a triplet multiplicity and the optimized structures and wavefunctions were subsequently used in BS-DFT calculations using the approach proposed by Noodleman as implemented in ORCA 5.0.4.[9,10] The exchange coupling constants calculated this way were –0.95 cm^{-1} (–28.5 GHz) and +0.38 cm^{-1} (11.4 GHz) for **$[(P3)_2\bullet(DABCO)_3]^{2-}$** and **$[(P3)_2\bullet(bipy)_3]^{2-}$**, respectively. These values are much larger than expected for these systems considering there is no covalent connection between the spin centers on the different porphyrin chains of the ladder complexes. In addition, it seems surprising that the calculations resulted in opposite signs for the interactions in the two different complexes. Many previous studies have reported difficulties in obtaining quantitative agreement between experimental and BS-DFT calculated exchange coupling values.[11,12,13] While there have been recent advances in BS-DFT approaches,[13,14] no further attempts at calculating J were made as it was possible to accurately simulate the spectra of the ladder complex dianions studied here even without the inclusion of any exchange interaction (as discussed above).

References

1 M. Hoffmann, J. Kärnbratt, M.-H. Chang, L. M. Herz, B. Albinsson, H. L. Anderson, *Angew. Chem. Int. Ed.* **2008**, *47*, 4993.

2 H.-I. Un, S. A. Gregory,S. K. Mohapatra, M. Xiong, E. Longhi, Y. Lu, S. Rigin, S. Jhulki, C.-Y. Yang, T. V. Timofeeva, J.-Y. Wang, S. K. Yee, S. Barlow, S. R. Marder, J. Pei, J., *Adv. Energy Mater.* **2019**, *9*, 1900817

3 S. Stoll, G. Jeschke, M. Willer, A. Schweiger, *J. Magn. Reson.* **1998**, *130*, 86.

4 O. A. Vydrov, G. E.Scuseria, *J. Chem. Phys.* **2006**, *125*, 234109.

5 C. Riplinger, J. P. Kao, G. M. Rosen, V. Kathirvelu, G. R. Eaton, S. S. Eaton, A. Kutateladze, F. Neese, *J. Am. Chem. Soc.* **2009**, *131*, 10092.

6 K. Ayabe, K. Sato, S. Nishida, T. Ise, S. Nakazawa, K. Sugisaki, Y. Morita, K. Toyota, D. Shiomi, M. Kitagawa, T. Takui, *Phys. Chem. Chem. Phys.* **2012**, *14*, 9137.

7 J. Hergenhahn, J. M. Holmes, J.-R. Deng, H. Gotfredsen,R. M. J. Jacobs, S. M. Kopp, C. R. Timmel, H. L. Anderson, *J. Am. Chem. Soc.* **2024**, *147*, 978.

8 H. J. Hogben, J. K. Sprafke, M. Hoffmann, M. Pawlicki, H. L. Anderson, *J. Am. Chem. Soc.* **2011**, *133*, 20962

9 F. Neese, *Comput. Mol. Sci.* **2022**, *12*, e1606.

10 L. Noodleman *J. Chem. Phys.* **1981**, *74*, 5737.

11 S. Richert, I. Kuprov, M. D. Peeks, E. A. Suturina, J. Cremers, H. L. Anderson, C. R. Timmel, *Phys. Chem. Chem. Phys.* **2017**, *19*, 16057.

12 G. Singh, S. Gamboa, M. Orio, D. A. Pantazis, M. Roemelt, *Theor. Chem. Acc.* **2021**, *140*, 139.

13 T. Quintes, M. Franz, P. Thielert, A. J. Redman, S. Richert*, Chem. Phys. Rev.* **2024**, *5*, 041312.

14 Y. Shi, Y. Shi, A.Wasserman, Y.Z. Li, *J. Chem. Theory Comput.* **2025**, *21*, 8420.
